# Blood product administration in the prehospital setting: a multisociety consensus statement

**DOI:** 10.1186/s44158-025-00248-9

**Published:** 2025-05-26

**Authors:** Luca Carenzo, Etrusca Brogi, Vanessa Agostini, Stefania Armani, Roberto Balagna, Maria Grazia Bocci, Antonio Cascio, Michela Ciminello, Andrea Cortegiani, Massimiliano Di Biagio, Patrizia Di Gregorio, Andrea Fabbri, Lara Gianesello, Guglielmo Imbriaco, Cristian Lupi, Lucia Mirabella, Stefano Paglia, Andrea Paoli, Silvia Pini, Silvano Rossini, Giovanni Sbrana

**Affiliations:** 1 Department of Anesthesia and Intensive Care Medicine, IRCCS Humanitas Research Hospital, Rozzano, Milano Italy; 2 Neuroscience Intensive Care Unit, ASST Grande Ospedale Metropolitano Niguarda, Milan, Italy; 3 Transfusion Department, IRCCS Ospedale Policlinico San Martino, Genoa, Italy; 4 Trentino Emergency Medical Services 118, Santa Chiara Hospital, Trento, Italy; 5 Anestesia E Rianimazione 3, ASL Di Torino, Turin, Italy; 6 Department of Anesthesia and Intensive Care, National Institute for Infectious Diseases L. Spallanzani IRCCS, Rome, Italy; 7 Department of Infectious and Tropical Diseases, Policlinico Paolo Giaccone, Palermo, Italy; 8 Department of Emergency and Urgent Care HEMS and Prehospital Emergency Services 118, Grosseto, Italy; 9 Department of Anesthesia, Intensive Care and Critical Care Medicine, Policlinico Paolo Giaccone, Palermo, Italy; 10 Department of Precision Medicine in Medical, Surgical and Critical Areas, University of Palermo, Palermo, Italy; 11 HEMS, ARES 118, Rome, Italy; 12 Ospedale SS Annunziata, Chieti, Roma, Italy; 13 Department of Emergency Medicine, Ospedale Morgagni-Pierantoni, AUSL Della Romagna, Forlì, Italy; 14 Department of Anesthesia and Intensive Care in Orthopedics, Careggi University Hospital, Florence, Italy; 15 118 Emergency Dispatch Center Emilia Est, Bologna, Italy; 16 Department of Anesthesia and Intensive Care, University Hospital Arcispedale Sant’Anna, Ferrara, Italy; 17 Department of Anesthesia and Intensive Care, University Hospital-University of Foggia, Foggia, Italy; 18 Department of Emergency Medicine, Ospedale Maggiore Di Lodi ASST Lodi, Lodi, Italy; 19 SUEM 118 Emergency Service, University Hospital of Padova, Padua, Italy; 20 Department of Anesthesia and Intensive Care, University Hospital of Pisa, Pisa, Italy; 21 Department of Immunohematology and Transfusion Medicine, ASST Grande Ospedale Metropolitano Niguarda, Milan, Italy

**Keywords:** Prehospital transfusion, Hemorrhagic shock, Blood products, Fibrinogen concentrate, SIAARTI, Emergency medical services, Clinical governance, Trauma care

## Abstract

The Italian Society of Anesthesia, Analgesia, Resuscitation and Intensive Care (SIAARTI) has led the development of a Good Clinical Practice (GCP) document, engaging multiple scientific societies—including the Italian Society of Transfusion Medicine and Immunohematology (SIMTI), the Italian Society of Infectious and Tropical Diseases (SIMIT), the Italian Society of Emergency Medicine (SIMEU), the National Association of Critical Care Nurses (ANIARTI), and the Italian National Blood Centre (Centro Nazionale Sangue-CNS). This collaborative effort aims to establish a multidisciplinary consensus on the administration of blood products in the prehospital management of patients with life-threatening hemorrhage. The increasing adoption of prehospital transfusion programs worldwide, particularly in trauma care, highlighted the need for structured recommendations that ensure safety, effectiveness, and compliance with current regulations. In prehospital settings, the early administration of packed red blood cells, fibrinogen concentrate, and fresh frozen plasma is not only considered feasible but has also shown to be potentially effective in improving hemodynamic stability and reducing mortality in patients with hemorrhagic shock. However, these benefits are strongly influenced by factors such as patient selection, timing of intervention, and the integration of transfusion protocols into advanced prehospital care systems. Implementing strict clinical governance, ensuring appropriate storage conditions, and developing standardized documentation processes are key to the success of these programs. Furthermore, close collaboration between emergency medical services and blood banks is essential to ensure compliance with national guidelines and to optimize patient outcomes. This consensus document was developed through a systematic literature review and a modified Delphi method, involving blind voting and consensus evaluation using a Likert scale. The process was conducted over two rounds of online voting. The document addresses four critical topics: the selection of blood product derivatives for prehospital use, safety requirements for their transport, documentation and traceability standards, and procedures for the return of unused components.

**Supplementary Information:**

The online version contains supplementary material available at 10.1186/s44158-025-00248-9.

## Background

Hemorrhagic shock is one of the leading causes of death in trauma patients in prehospital settings. In cases of traumatic shock, early hemorrhage control and effective volume resuscitation with blood products are essential life-saving interventions [1–3].

Reflecting this, the transfusion of blood components such as red blood cells (RBCs) and plasma has become increasingly common in prehospital and transport medicine [4]. It is known from hospital-based studies that delays in initial blood product administration are associated with prolonged time to achieve hemostasis and an increase in mortality [5, 6].

In this context, the potential benefits of out-of-hospital administration of whole blood or blood products such as fibrinogen and prothrombin complex concentrate in select patients are being investigated. Although the increasing practice of out-of-hospital transfusion (OHT) suggests consensus on a likely clinical benefit, evidence regarding the effect of OHT on morbidity and mortality is limited and conflicting [7–9]. Moreover, the generalizability of the limited evidence is further complicated by regional variability in geography, patient populations, and healthcare system organization, all of which influence the feasibility and effectiveness of OHT.

More recently, the inclusion of blood products within prehospital advanced resuscitative care protocols—comprising calcium and tranexamic acid administration—for patients with life-threatening hemorrhage due to penetrating trauma in high-intensity urban settings has demonstrated a survival benefit compared to standard care without blood products [8]. It is important to note that out-of-hospital management of acute hemorrhage extends beyond OHT and includes factors such as administration of tranexamic acid, avoidance of hypothermia and physical means of hemorrhage control where possible. Efficient and effective implementation of OHT requires a combination of medical and logistic considerations that span multiple specialties.

In Italy, since 2020, some Helicopter Emergency Medical Services (HEMS) in the regions of Emilia-Romagna, Tuscany, Lombardy, and Apulia have initiated prehospital transfusion programs involving blood products [10, 11]. Although the long-term impact on mortality remains under investigation through multicenter studies, preliminary data from these Italian experiences confirm both the technical feasibility and the logistical manageability of prehospital transfusion programs [3, 10].

Nonetheless, there is still a lack of comprehensive guidance supporting the implementation of prehospital transfusion systems in compliance with current legislation, with particular emphasis on clinical governance and the safe management of blood products in this specific setting. The implementation of such programs is often perceived by healthcare institutions (hospitals and operational units) as a complex and high-risk process, both from a legal and procedural standpoint.

To address these challenges, we invited an expert panel to develop national consensus recommendations on out-of-hospital hemorrhage management and OHT. These guidelines aim to support the safe and effective implementation of OHT in Italy, providing a structured framework to optimize patient outcomes and system integration.

## Methods

The methodology adopted for this document is in line with the current regulations [12] of the Italian Society of Anesthesia, Analgesia, Resuscitation and Intensive Care (SIAARTI) for the development of Good Clinical Practice documents through a consensus process. This Good Clinical Practice document was developed by a multidisciplinary panel led by SIAARTI, with the participation of the Italian Society of Transfusion Medicine and Immunohematology (SIMTI), the Italian Society of Infectious and Tropical Diseases (SIMIT), the Italian Society of Emergency Medicine (SIMEU), the National Association of Critical Care Nurses (ANIARTI), and the Italian National Blood Centre (Centro Nazionale Sangue-CNS).

The methodological pathway for this type of Good Clinical Practice document, as outlined in the SIAARTI regulations, was designed by an anesthesiologist with expertise in methodology (A.C.) and was based on the principles of systematic literature review and the modified Delphi method [13].

### Scope and target audience

The scope of this document is to provide guidance on the prehospital administration of blood products in patients with life-threatening hemorrhage. Specifically, it aims to outline the organizational modalities and clinical governance principles necessary for the implementation of safe and effective prehospital transfusion programs, ensuring compliance with current legislation.

It aims to support the implementation of safe and effective prehospital transfusion programs in compliance with current legislation and clinical governance principles.

The target audience includes physicians, critical care nurses, emergency medical services (EMS) personnel, transfusion medicine specialists, and policymakers involved in prehospital trauma care and transfusion services.

### Panel selection and organization

The proposal for this document was submitted by the document coordinator (G.S.) in response to an initial public call for proposals seeking project ideas for the development of society-endorsed guidelines. Subsequently, the proposal was evaluated and approved by the SIAARTI Board of Directors, which selected the expert panel members based on their proven clinical and scientific experience in the relevant field.

On October 4 th, 2024, a multidisciplinary and intersocietal working group (WG) was formally established, composed of experts selected for their specific competencies in the area of interest. The panel consisted of 18 experts, appointed by the SIAARTI Board of Directors based on their curricula vitae and professional qualifications.

The selection and appointment process involved the participation of major national scientific societies relevant to the specific specialties. In particular, the following societies contributed to the process:

- The Italian Society of Infectious and Tropical Diseases (SIMIT)
- The Italian National Blood Centre of the Italian National Institute of Health (CNS)
- The National Association of Critical Care Nurses (ANIARTI)
- The Italian Society of Transfusion Medicine and Immunohematology (SIMTI)
- The Italian Society of Emergency Medicine (SIMEU)

Additionally, an expert in search strategy was involved to oversee the systematic review of the literature.

### Items selection

During the initial meeting, the methodology was shared with the panel, and topics were assigned to specific members according to their areas of expertise. The coordinators proposed four key items, which were then discussed during an online scoping workshop and prioritized through a structured voting process. The items focused on the use of blood products in the prehospital settings, the safety requirements for their transport, the documentation needed to ensure traceability of prehospital transfusions, and the procedures for the return and management of unused blood products. Each item was assigned to one or more panel members based on their expertise. Panelists were responsible for reviewing the available scientific literature and drafting statements, accompanied by supporting rationale in explanatory text format.

### Literature search

A systematic literature review was performed using PubMed, covering the period from 2013 to April 12 th, 2023. The search strategy incorporated both MeSH terms and free-text keywords, detailed in Supplementary Table 1.

We included original articles, narrative reviews, systematic reviews, meta-analyses, position papers, guidelines, experimental studies, and randomized controlled trials, as these represent the most informative and methodologically robust sources for evidence synthesis. We excluded non-English language articles due to the lack of resources for accurate translation and critical appraisal, a limitation acknowledged in PRISMA. We also excluded conference proceedings, case reports, case series, animal studies, and pediatric studies, as these typically provide limited generalizability or are outside the scope of our review objective. The literature search and reporting adhered to the PRISMA 2020 guidelines [14].

### Statements formulation

Based on the literature review and their own expertise, panelists drafted statements and rationales which were then subjected to a blind voting process, excluding the methodologist (L.M.). The consensus process followed a modified Delphi method and allowed for a maximum of two online voting rounds.

Each panelist rated the appropriateness of each statement using an ordinal Likert scale, according to the RAND/UCLA method [15], with scores ranging from 1 to 9:

- 1–3: Inappropriate (disagreement)
- 4–6: Uncertain
- 7–9: Appropriate (agreement)

During the first voting round, panelists were invited to provide free-text comments and suggestions to enrich the evaluation process.

Predefined consensus criteria required:

1. At least 75% of participants to assign a score within the same interval (1–3, 4–6, or 7–9).
2. The median score to fall within the same interval.

Consensus was classified based on the position of the median score, allowing the identification of unanimous consensus, partial consensus, or significant disagreement. A second voting round was not required, as all statements reached consensus in the first round. The results of the voting process were summarized in tabular form, detailed in Supplementary Tables S2 and S3. Two external reviewers (L.C., A.C.) reviewed the statements and provided feedback that was discussed with the panel and incorporated into the final version of the manuscript.

### Conflict of interest (COI) management

All panelists were required to disclose any potential intellectual or financial conflicts of interest (COI) that could have influenced their involvement in the project. Each panelist completed a formal declaration in accordance with SIAARTI procedures. No conflicts of interest were declared by any member of the panel.

Panelists did not receive any financial incentives for their participation in the development of this Good Clinical Practice (GCP) document. Furthermore, no funding or input from industry stakeholders was incorporated into the process or content of the document.

## Results

### Blood products administration in the prehospital setting

Question: Which blood products should be used in the prehospital settings?

#### Statement 1.1

The panel believes that blood products—including packed red blood cells (PRBCs), fresh frozen plasma (FFP), fibrinogen concentrate (FC), and plasma derivatives such as coagulation factors—can be safely transported and administered in prehospital environments. It is the responsibility of each healthcare organization, through its clinical governance evaluation process, to define which components and derivatives should be prioritized according to its specific organizational structure and needs.

#### Statement 1.2

Based on Italian experience and the organization of transfusion services within the country, the panel considers the prehospital use of packed red blood cells a valid option in terms of effectiveness, feasibility, transportability, and storage.

#### Statement 1.3

In patients experiencing hemorrhagic shock, the administration of fibrinogen concentrate, in addition to PRBCs, is considered feasible in prehospital settings, following protocols described in the current literature [16–18].

#### Background

Extensive and well-established experience from North American, European, and Italian pilot studies supports the prehospital use of blood products, recognizing their potential to improve clinical outcomes in critically bleeding patients [19–23]. Current evidence demonstrates the technical feasibility and safety of prehospital transfusion procedures [4, 24–27].

According to the Italian National Institute of Health (Istituto Superiore di Sanità, ISS) guidelines on integrated management of major trauma from the scene to definitive care, the prehospital transfusion of blood products is recommended in hemorrhaging trauma patients whenever feasible (Recommendation 24). Early transfusion contributes not only to volume expansion but also plays a key role in the prompt treatment of hemorrhagic shock and trauma-induced coagulopathy—two critical factors in preventing mortality.

Despite the low quality of evidence, the panel of the ISS guidelines emphasizes that the implementation of prehospital transfusion programs should be a top priority for healthcare systems. This recommendation is based on solid pathophysiological principles and recent scientific evidence, highlighting the added value of an integrated and advanced trauma management approach.

The document acknowledges the logistical and organizational challenges associated with the prehospital use of blood products but underlines its strategic importance within a highly medicalized emergency care system. This requires careful planning and active involvement of transfusion services in defining shared protocols, particularly concerning storage, transport, and the management of unused stocks. In conclusion, early transfusion of blood products is recommended as a crucial intervention to improve outcomes in patients with major trauma.

In terms of which blood products can be transported and administered prehospital, Italian pilot programs have demonstrated the feasibility and utility of all major components. Typically, carrying two units of PRBCs is considered adequate for most centers, while three units may be preferable for centers covering large rural or mountainous areas where stabilization and transfer times are prolonged. Critically injured patients derive the most benefit from the availability of multiple units.

The concept of “hemostatic resuscitation” emphasizes the early support of coagulation through the use of blood products, plasma derivatives, and pharmacologic agents. Plasma—either fresh frozen (FFP) or lyophilized—is often used to complement PRBCs in supporting coagulation. However, plasma presents logistical challenges, particularly in prehospital settings. While it serves as an effective volume expander and is part of 1:1:1 resuscitation protocols, it does not provide sufficient fibrinogen to correct the deficit associated with hemorrhagic shock [28].

Fibrinogen plays a critical role in clot formation, and its rapid depletion in trauma patients is associated with increased mortality [29]. Fibrinogen replacement can be achieved through FFP or fibrinogen concentrate (FC). Collins’ theoretical model allows for comparison of the units or vials required and the volume needed to increase plasma fibrinogen levels by approximately 1 g/L [30].

Current data highlight the advantages of FC over FFP, particularly in prehospital contexts where speed and operational simplicity are essential. Early fibrinogen replacement has been shown to improve outcomes in severe trauma [31]. FC offers multiple benefits compared to traditional plasma derivatives like cryoprecipitate, including ambient temperature storage and rapid preparation, making it ideal for prehospital use [32].

Clinical trials have demonstrated that FC reduces the need for PRBC transfusions and maintains a high safety profile, supported by more than three decades of use in Europe [33–35]. While high-quality clinical evidence is still limited, European guidelines for the management of major bleeding and coagulopathy recommend both FC and cryoprecipitate for fibrinogen replacement [16].

In prehospital settings, FC represents a particularly advantageous option due to its speed and simplicity of administration. It is considered an effective, safe, and logistically favorable intervention with the potential to significantly improve outcomes in patients with life-threatening hemorrhage.

Multiple formulations are available that ensure safe transportation and storage in prehospital environments. The working group is also monitoring potential future developments in the use of low-titer group O whole blood (LTOWB), which is not currently available in Italy.

### Safety requirements for the prehospital transport of blood products

Question: What are the safety requirements for the prehospital transport of blood products?

#### Statement 2.1

The panel recommends that advanced prehospital care teams are responsible for maintaining the correct storage temperature of blood products and ensuring proper traceability of transfusions when performed.

#### Statement 2.2

The panel recommends that, in collaboration with the reference transfusion services, systems must be in place to guarantee the continuous measurement and recording of storage temperatures for transportation devices throughout the entire transport duration.

#### Statement 2.3

The panel recommends that blood product containers should always be onboard rescue vehicles, just like any other standard emergency equipment.

#### Background

Most studies report the use of thermal containers capable of maintaining appropriate storage temperatures for 48–72 h [36–38]. Temperature monitoring can be performed on either the container or the individual blood bags, using data loggers, colorimetric indicators, or electronic probes connected to smartphones. These systems provide alerts if the temperature rises above safe levels, compromising the preservation of the components.

Commercially available systems currently ensure the safe and stable transportation of blood products, complying with service, safety, and regulatory requirements [39, 40]. Such containers can remain onboard emergency vehicles even when exposed to extreme environmental conditions, guaranteeing the thermal and physical stability of the components.

The working group recommends planned container replacement every 24 h to maintain an operational safety margin in case of prolonged unexpected activations (a common scenario in helicopter EMS). Thermal and microbiological monitoring procedures should be jointly defined between prehospital services and transfusion centers. Preparation of the containers can be carried out by either the transfusion services or the prehospital teams, provided that personnel are adequately trained and procedures are standardized.

From an organizational perspective, the working group estimates that the prehospital transport of blood products is feasible in at least 60–70% of centers, provided they comply with current regulations governing packaging and transport to maintain product integrity and biological characteristics.

Discard rates for blood products reported in international studies range from 0.5 to 1.9% [37, 41–45]. The Italian experience across four helicopter EMS bases since 2020 has reported no wastage.

### Documentation required to ensure traceability of prehospital transfusions

Question: What documentation is required to ensure traceability of prehospital transfusions?

#### Statement 3.1

The panel recommends that documentation of transfused blood products and the recipient’s clinical condition before and after transfusion should be included in the patient’s clinical records, in compliance with transfusion regulations.

#### Statement 3.2

The panel recommends that each transfusion center adopt, or develop, an analog or preferably digital system to ensure traceability of the urgent assignment of transfused blood products in prehospital settings.

#### Background

Traceability documentation for prehospital transfusions must comply with current regulations [46–49]. Specifically, the following elements must be ensured:

1. A request form for urgent blood products, according to the digital or analog system in use
2. A delivery form detailing the information on each blood product unit provided
3. A label on each unit indicating its identification data
4. Transfusion records included in the recipient’s clinical documentation (paper or electronic), specifying:
  ◦ Number, type, and identification code of each unit transfused
  ◦ Start and end time of the transfusion
  ◦ Vital parameters recorded at the start and within 60 min after completion
  ◦ Any adverse reactions and their management
5. A declaration of transfusion and any adverse reactions, reported to the transfusion service using designated forms (analog or digital)
6. Reporting of adverse events as required by current legislation

A univocal system for patient/component association should be implemented to ensure accurate and safe traceability (e.g., color-coded wristbands for each unit).

The working group recommends using a wristband attached to the recipient to assist in situations where blood products may be transfused to more than one unidentified patient during the same intervention, a rare but possible event in prehospital emergencies.

### Return of unused blood products

Question: When and how should unused blood products be returned?

#### Statement 4.1

The panel recommends that the return of unused blood products be carried out according to timeframes agreed upon with the reference transfusion service.

#### Statement 4.2

The panel recommends that blood products be returned to the transfusion service in all cases where correct storage temperature has not been maintained.

#### Statement 4.3

The panel recommends that all returned units be accompanied by documentation certifying integrity, proper storage, and transportation conditions.

#### Background

Returned blood products should:

1. Be returned according to the schedule established with the transfusion service to avoid returning units close to expiry
2. Be returned whenever the container or blood bank fails to maintain the required storage temperature; the duration of any temperature deviation should be reported to the transfusion service
3. Be returned whenever any component has been partially transfused, leading to the breaking of the container seal (when applicable)

All returned units must be accompanied by documentation certifying their integrity and compliance with storage and transport procedures as defined by the local Hospital Transfusion Committee.

## Conclusions

In conclusion, this Good Clinical Practice (GCP) document provides a series of structured recommendations, developed through a rigorous methodological process, for the administration and management of blood products in the prehospital setting. The expert panel produced 11 statements, based on the available scientific literature and supported by current national and international guidelines.

The document specifically addresses four key areas:

- The use of blood products in prehospital settings,
- The safety requirements for their transport,
- The documentation necessary to ensure traceability of transfusions,
- And the procedures for the return and management of unused blood products.

These recommendations are intended to support healthcare organizations and prehospital emergency services in developing standardized, safe, and effective transfusion protocols, ensuring compliance with regulatory requirements and promoting clinical governance in this complex and critical area of patient care.

## Supplementary Information

Supplementary Material 1. Table S1: Search Strategy and PRISMA Flow Diagram.

Supplementary Material 2. Table S2: Voting Results: ITEM–First Round.

Supplementary Material 3. Table S3: Voting Results: Statements and Rationale.

## Data Availability

No datasets were generated or analysed during the current study.
