# Supplementary material for: Blood product administration in the prehospital setting: a multisociety consensus statement
Source: J Anesth Analg Crit Care. 2025 May 26;5:28. doi: 10.1186/s44158-025-00248-9 (PMC12105163; doi:10.1186/s44158-025-00248-9)
Supplement: Supplementary file 1 — Supplementary Material 1. Table S1: Search Strategy and PRISMA Flow Diagram. [file 44158_2025_248_MOESM1_ESM.docx]

**Table 1 – Search Strategy and PRISMA Flow Diagram**

**Search strategy**

The systematic literature search was conducted using PubMed. The search strategy employed the following terms and keywords:

("blood" AND ("derivatives" OR "components") AND ("pre-hospital" OR "prehospital")) AND ("prehospital transfusion" OR "fibrinogen in prehospital setting" OR "integrity of hemocomponents" OR "equipped blood banks for transport" OR "hemocomponent storage" OR "hemocomponents not used documents" OR "tranexamic acid in prehospital setting") AND ("2013" : "2023")

**PRISMA 2020 flow diagram for new systematic reviews which included searches of databases and registers only**

**Identification of studies via databases and registers**

Records identified from*:

Databases (n =1)

Registers (n =0)

Records removed *before screening*:

Duplicate records removed (n =27)

**Identification**

Records excluded**

(n = 13)

Records screened

(n =223)

Reports not retrieved

(n =10)

Reports sought for retrieval

(n = 210)

**Screening**

Reports excluded☹n=40)

PDF non accessible (n = 32)

Pediatric study (n =5)

Other languages (n = 3)

Reports assessed for eligibility

(n =200)

Studies included in review

(n = 160)

Reports of included studies

(n =30)

**Included**

Source: Page MJ, et al. BMJ 2021;372:n71. doi: 10.1136/bmj.n71.

# This work is licensed under CC BY 4.0. To view a copy of this license, visit <https://creativecommons.org/licenses/by/4.0/>
