# Supplementary material for: Blood product administration in the prehospital setting: a multisociety consensus statement
Source: J Anesth Analg Crit Care. 2025 May 26;5:28. doi: 10.1186/s44158-025-00248-9 (PMC12105163; doi:10.1186/s44158-025-00248-9)
Supplement: Supplementary file 2 — Supplementary Material 2. Table S2: Voting Results: ITEM–First Round. [file 44158_2025_248_MOESM2_ESM.docx]

**Table 2 - Voting Results: ITEM – First Round**

|  | **What are the safety requirements for the transport of blood components?** | ***Which blood products should be used in the prehospital settings?*** | ***When and how should unused blood products be returned?*** | ***What documentation is required to ensure traceability of prehospital transfusions?*** | **What are the emergency transfusion clinical indications for packed red blood cells and other blood components? The indications for the use of blood derivatives in the prehospital setting may be the same as those used to trigger the in-hospital massive transfusion protocol?** |
| --- | --- | --- | --- | --- | --- |
| #1 | 7 | 6 | 7 | 6 | 4 |
| #2 | 7 | 7 | 7 | 6 | 5 |
| #3 | 9 | 8 | 8 | 8 | 5 |
| #4 | 9 | 9 | 8 | 8 | 6 |
| #5 | 9 | 9 | 8 | 9 | 6 |
| #6 | 9 | 9 | 8 | 9 | 6 |
| #7 | 9 | 9 | 9 | 9 | 7 |
| #8 | 9 | 9 | 9 | 9 | 8 |
| #9 | 9 | 9 | 9 | 9 | 8 |
| #10 | 9 | 9 | 9 | 9 | 9 |
| #11 | 9 | 9 | 9 | 9 | 9 |
| #12 | 9 | 9 | 9 | 9 | 9 |
| #13 | 9 | 9 | 9 | 9 | 9 |
| #14 | 9 | 9 | 9 | 9 | 9 |
| #15 | 9 | 9 | 9 | 9 | 9 |
| #16 | 9 | 8 | 9 | 9 | 9 |
| #17 | 9 | 9 | 9 | 9 | 9 |
| **Agreement** | **100%** | **94.11%** | **100%** | **88.2%** | **64.7%** |
|  |  |  |  |  |  |
| Minimum | 7 | 6 | 7 | 6 | 4 |
| Median | 9 | 9 | 9 | 9 | 8 |
| Maximum | 9 | 9 | 9 | 9 | 9 |
