# Supplementary material for: Blood product administration in the prehospital setting: a multisociety consensus statement
Source: J Anesth Analg Crit Care. 2025 May 26;5:28. doi: 10.1186/s44158-025-00248-9 (PMC12105163; doi:10.1186/s44158-025-00248-9)
Supplement: Supplementary file 3 — Supplementary Material 3. Table S3: Voting Results: Statements and Rationale. [file 44158_2025_248_MOESM3_ESM.docx]

# Table 3 - Voting Results: Statements and Rationale

| 1. **Which blood products should be used in the prehospital settings?** | | | | |
| --- | --- | --- | --- | --- |
|  | **Statement 1.1**  The panel believes that all blood products—including packed red blood cells (PRBCs), fresh frozen plasma (FFP), fibrinogen concentrate (FC), and plasma derivatives such as coagulation factors—can be safely transported and administered in prehospital environments. It is the responsibility of each healthcare organization, through its clinical governance evaluation process, to define which components and derivatives should be prioritized according to its specific organizational structure and needs. | **Statement 1.2**  Based on Italian experience and the organization of transfusion services within the country, the panel considers the prehospital use of packed red blood cells a valid option in terms of effectiveness, feasibility, transportability, and storage. | **Statement 1.3**  In patients experiencing haemorrhagic shock, the administration of fibrinogen concentrate, in addition to PRBCs, is considered feasible in prehospital settings, following protocols described in the current literature | **Statement 1.4**  The panel believes that, in the case of a patient in extremis due to haemorrhagic trauma (peri-arrest condition), the infusion of packed red blood cells, administration of tranexamic acid and fibrinogen—possibly in combination with the use of REBOA—should be considered, as recommended by the Italian National Institute of Health (ISS) guidelines for trauma management. |
| #1 | 6 | 7 | 7 | 6 |
| #2 | 6 | 7 | 8 | 7 |
| #3 | 7 | 8 | 8 | 7 |
| #4 | 7 | 9 | 9 | 7 |
| #5 | 7 | 9 | 9 | 7 |
| #6 | 8 | 9 | 9 | 8 |
| #7 | 9 | 9 | 9 | 8 |
| #8 | 9 | 9 | 9 | 8 |
| #9 | 9 | 9 | 9 | 9 |
| #10 | 9 | 9 | 9 | 9 |
| #11 | 9 | 9 | 9 | 9 |
| #12 | 9 | 9 | 9 | 9 |
| #13 | 9 | 9 | 9 | 9 |
| #14 | 9 | 9 | 9 | 9 |
| #15 | 9 | 9 | 9 | 9 |
| #16 | 9 | 9 | 9 | 9 |
| #17 | 9 | 9 | 9 | 9 |
| #18 | 9 | 9 | 9 | 9 |
| **Agreement** | 16/18 | 18/18 | 18/18 | 17/18 |
| **Agreement %** | 88.80% | 100% | 100% | 94.40% |
|  |  |  |  |  |
| **Minimum** | 6 | 7 | 7 | 6 |
| **Quartile 1** | 7.25 | 9 | 9 | 7.25 |
| **Median** | 9 | 9 | 9 | 9 |
| **Quartile 3** | 9 | 9 | 9 | 9 |
| **Maximum** | 9 | 9 | 9 | 9 |

| 1. **What are the safety requirements for the prehospital transport of blood products?** | | | |
| --- | --- | --- | --- |
|  | Statement 2.1  The panel recommends that advanced prehospital care teams are responsible for maintaining the correct storage temperature of blood products and ensuring proper traceability of transfusions when performed. | Statement 2.2  The panel recommends that, in collaboration with the reference transfusion services, systems must be in place to guarantee the continuous measurement and recording of storage temperatures for transportation devices throughout the entire transport duration. | Statement 2.3  The panel recommends that blood product containers should always be onboard rescue vehicles, just like any other standard emergency equipment. |
| #1 | 7 | 7 | 7 |
| #2 | 7 | 7 | 9 |
| #3 | 8 | 8 | 9 |
| #4 | 9 | 9 | 9 |
| #5 | 9 | 9 | 9 |
| #6 | 9 | 9 | 9 |
| #7 | 9 | 9 | 9 |
| #8 | 9 | 9 | 9 |
| #9 | 9 | 9 | 9 |
| #10 | 9 | 9 | 9 |
| #11 | 9 | 9 | 9 |
| #12 | 9 | 9 | 9 |
| #13 | 9 | 9 | 9 |
| #14 | 9 | 9 | 9 |
| #15 | 9 | 9 | 9 |
| #16 | 9 | 9 | 9 |
| #17 | 9 | 9 | 9 |
| #18 | 9 | 9 | 9 |
| Agreement | 18/18 | 18/18 | 18/18 |
| Agreement % | 100% | 100% | 100% |
|  |  |  |  |
| Minimum | 7 | 7 | 3 |
| Quartile 1 | 9 | 9 | 9 |
| Median | 9 | 9 | 9 |
| Quartile 3 | 9 | 9 | 9 |
| Maximum | 9 | 9 | 9 |

|  | **3.What documentation is required to ensure traceability of prehospital transfusions?** | | **4.When and how should unused blood products be returned?** | | |
| --- | --- | --- | --- | --- | --- |
|  | Statement 3.1  The panel recommends that documentation of transfused blood products and the recipient’s clinical condition before and after transfusion should be included in the patient’s clinical records, in compliance with transfusion regulations. | Statement 3.2  The panel recommends that each transfusion center adopt, or develop, an analog or preferably digital system to ensure traceability of the urgent assignment of transfused blood products in prehospital settings. | Statement 4.1  The panel recommends that the return of unused blood products be carried out according to timeframes agreed upon with the reference transfusion service. | Statement 4.2  The panel recommends that blood products be returned to the transfusion service in all cases where correct storage temperature has not been maintained. | Statement 4.3  The panel recommends that all returned units be accompanied by documentation certifying integrity, proper storage, and transportation conditions. |
| #1 | 9 | 7 | 6 | 6 | 8 |
| #2 | 9 | 7 | 7 | 8 | 9 |
| #3 | 9 | 9 | 8 | 9 | 9 |
| #4 | 9 | 9 | 8 | 9 | 9 |
| #5 | 9 | 9 | 9 | 9 | 9 |
| #6 | 9 | 9 | 9 | 9 | 9 |
| #7 | 9 | 9 | 9 | 9 | 9 |
| #8 | 9 | 9 | 9 | 9 | 9 |
| #9 | 9 | 9 | 9 | 9 | 9 |
| #10 | 9 | 9 | 9 | 9 | 9 |
| #11 | 9 | 9 | 9 | 9 | 9 |
| #12 | 9 | 9 | 9 | 9 | 9 |
| #13 | 9 | 9 | 9 | 9 | 9 |
| #14 | 9 | 9 | 9 | 9 | 9 |
| #15 | 9 | 9 | 9 | 9 | 9 |
| #16 | 9 | 9 | 9 | 9 | 9 |
| #17 | 9 | 9 | 9 | 9 | 9 |
| #18 | 9 | 9 | 9 | 9 | 9 |
| Agreement | 18/18 | 18/18 | 17/18 | 17/18 | 18/18 |
| Agreement % | 100% | 100% | 94.40% | 94.40% | 100% |
|  |  |  |  |  |  |
| Minimum | 9 | 7 | 6 | 6 | 8 |
| Quartile 1 | 9 | 9 | 9 | 9 | 9 |
| Median | 9 | 9 | 9 | 9 | 9 |
| Quartile 3 | 9 | 9 | 9 | 9 | 9 |
| Maximum | 9 | 9 | 9 | 9 | 9 |
